# Development of a tool to measure person-centered maternity care in developing settings: validation in a rural and urban Kenyan population

**DOI:** 10.1186/s12978-017-0381-7

**Published:** 2017-09-22

**Authors:** Patience A. Afulani, Nadia Diamond-Smith, Ginger Golub, May Sudhinaraset

**Affiliations:** 1 School of Medicine, University of California, San Francisco, USA; 2 Innovations for Poverty Action, Nairobi, Kenya; 3 Fielding School of Public Health, University of California, Los Angeles, USA

**Keywords:** Person-centered care, Maternity care, Measurement, Validation, Developing settings, Sub-Saharan Africa, Kenya

## Abstract

**Background:**

Person-centered reproductive health care is recognized as critical to improving reproductive health outcomes. Yet, little research exists on how to operationalize it. We extend the literature in this area by developing and validating a tool to measure person-centered maternity care. We describe the process of developing the tool and present the results of psychometric analyses to assess its validity and reliability in a rural and urban setting in Kenya.

**Methods:**

We followed standard procedures for scale development. First, we reviewed the literature to define our construct and identify domains, and developed items to measure each domain. Next, we conducted expert reviews to assess content validity; and cognitive interviews with potential respondents to assess clarity, appropriateness, and relevance of the questions. The questions were then refined and administered in surveys; and survey results used to assess construct and criterion validity and reliability.

**Results:**

The exploratory factor analysis yielded one dominant factor in both the rural and urban settings. Three factors with eigenvalues greater than one were identified for the rural sample and four factors identified for the urban sample. Thirty of the 38 items administered in the survey were retained based on the factors loadings and correlation between the items. Twenty-five items load very well onto a single factor in both the rural and urban sample, with five items loading well in either the rural or urban sample, but not in both samples. These 30 items also load on three sub-scales that we created to measure dignified and respectful care, communication and autonomy, and supportive care. The Chronbach alpha for the main scale is greater than 0.8 in both samples, and that for the sub-scales are between 0.6 and 0.8. The main scale and sub-scales are correlated with global measures of satisfaction with maternity services, suggesting criterion validity.

**Conclusions:**

We present a 30-item scale with three sub-scales to measure person-centered maternity care. This scale has high validity and reliability in a rural and urban setting in Kenya. Validation in additional settings is however needed. This scale will facilitate measurement to improve person-centered maternity care, and subsequently improve reproductive outcomes.

**Electronic supplementary material:**

The online version of this article (10.1186/s12978-017-0381-7) contains supplementary material, which is available to authorized users.

## Plain English summary

High maternal mortality remains a pressing problem in developing settings. Poor person-centered maternity care contributes both directly and indirectly to this problem. Person-centered maternity care refers to care during childbirth that is respectful and responsive to individual women and their families’ preferences, needs, and values. Person-centered maternity care emphasizes the quality of patient experience. Although experts in maternal health recognize the importance of person-centered maternity care, a consistent way to measure this construct has not yet been developed. We hope to address this problem by presenting a tool to measure person-centered maternity care. In this paper, we describe the process of developing the tool, and the analysis to test whether the tool measures what it is intended to measure consistently. Our analysis shows that the tool that we developed—comprised of 30 questions—is an effective tool to measure person-centered maternity care in both rural and urban settings in Kenya. The tool includes questions to measure dignity and respect, communication and autonomy, and supportive care. This tool is likely useful in other developing settings, although more testing is required in additional settings. The tool can be used for research to identify the factors that affect person-centered maternity care, as well as its consequences. Program planners may also use this tool to identify which aspects of person-centered maternity care need attention, and to assess if interventions lead to improvements in women’s experiences during childbirth.

## Background

Despite progress in reducing maternal mortality, developing regions still account for approximately 99% of global maternal deaths, with sub-Saharan Africa accounting for roughly 66% of these deaths [1]. Historically, limited access to maternal health services has contributed significantly to adverse maternal outcomes [2–4]. However, increases in use of maternal health services over the past decade has not been matched with reductions in maternal mortality, exposing a crucial gap in quality of care [5–7]. This has increased the momentum for improving quality of maternal and reproductive health care in the last few years, with calls for a more comprehensive focus on quality of care—beyond provision of essential services solely [8–10].

Recent evidence of poor treatment of women during childbirth and related calls to action have increased attention on poor person-centered care during childbirth in developing settings [5, 10]. Although these calls to action utilize positive terminology such as respectful and dignified maternity care [11, 12], the terminology used to describe the growing body of research on this topic has largely been negative, with terms like “disrespect and abuse” [13–17], “obstetric violence” [18, 19], “dehumanized care” [20, 21], and “mistreatment of women” [22, 23]. A landscape analysis by Bowser and Hill in 2010 described seven manifestations of disrespect and abuse of women during childbirth. These included physical abuse, non-dignified care, non-consented care, non-confidential care, discrimination, abandonment, and detainment [14]. In a mixed methods systematic review, Bohren et al. (2015) also identified seven domains of mistreatment commonly described in the qualitative literature, including physical abuse, sexual abuse, verbal abuse, stigma and discrimination, failure to meet professional standards of care, poor rapport between women and providers, and health system conditions and constraints [22]. This review also highlighted the lack of standardized quantitative measures to evaluate women’s experience during childbirth [22].

Person-Centered Maternity Care (PCMC) highlights respectful maternity care as part of the broader interest in person-centered care. PCMC expands the discussion beyond poor treatment to emphasize holistic, responsive, and dignified maternity care. PCMC emphasizes experience of care, and includes dimensions such as communication, respect and dignity, and emotional support, which are highlighted in the World Health Organization (WHO) quality of care framework for maternal and newborn health [24]. It is these person-centered dimensions that most often influence patients’ perceptions of quality of care and satisfaction with services [25–30]. Moreover, patients’ perceptions of quality of care indicate how well health systems meet patients’ expectations, as well as their trust in the system [30, 31]. These person-centered dimensions also affect clinical outcomes [32]. A recent systematic review found that patient experience was positively associated with clinical effectiveness and safety in more than 75% of published studies [33]. Additionally, person-centered care affects demand for services [34–36].

There is increasing evidence of poor PCMC in Kenya—potentially contributing to low facility delivery rates and a high maternal mortality rate. In 2015, the maternal mortality ratio for Kenya was estimated to be 510 deaths per 100,000 live births [1]. The most recent Kenyan Demographic and Health Survey (2014) found that 62% of women who had a baby in the previous 5 years delivered in a health facility, although wide disparities exists, especially as related to socioeconomic status [37]. For example, approximately 25% of women with no education and 31% of women in the lowest wealth quintile delivered in health facilities, compared to 85% of women with secondary or higher education and 93% among those in the highest wealth quintile respectively) [37]. Disparities in PCMC likely contribute to these disparities in facility deliveries [38]. Past research in Kenya has found that fear of receiving undignified care was one of the primary reasons for women choosing not to deliver in a facility [39]. Given the introduction of free maternity services in Kenya that reduce financial barriers to accessing care [40], perceptions of poor PCMC may be accounting for a larger proportion of the disparities in facility deliveries. A recent survey of women leaving postnatal wards in Kenya found that 20% of women reported some form of mistreatment, primarily non-dignified care, neglect or abandonment, non-confidential care, and detainment for not paying fees [13]. Another recent study in Kenya showed poor quality of antenatal and delivery care in many facilities in Kenya, with poor women being more likely to receive poor quality care: only 17% of all women and 8% of poor women had access to minimally adequate delivery care [41].

Poor PCMC has multiplicative effects, as it can directly lead to poor pregnancy outcomes, in addition to decreased demand for services [5, 10, 42]. It results in delayed, inadequate, unnecessary, or harmful care, minimizing the opportunity for health gains for both mothers and babies [10]. PCMC therefore needs more emphasis as a valued quality domain, as well as an indicator of human rights [5, 12, 11]. Despite growing evidence of its importance, there is a lack of consensus on how to operationalize PCMC. Most studies on mistreatment of women have been qualitative [22], and the few quantitative studies use binary measures [13, 43]. To our knowledge, only one published study has validated a tool to measure perceptions of respectful maternity care in a developing setting using standard procedures for scale development including psychometric analysis [44]. Without standardized and validated tools, the momentum behind measuring and improving PCMC could stagnate due to lack of clarity in what constitutes PCMC, and how best to target focused intervention efforts. There is therefore an urgent need to develop and validate tools to assess PCMC that can be used across multiple developing contexts. We aim to extend the literature by developing and validating a person-centered maternity care scale. This tool will have both research and programmatic utility. It will be useful for research to understand the determinants and consequences of poor PCMC, and will help health programs and providers to develop and target interventions. In addition, the tool has the potential to be used for future needs assessments, as well as monitoring and evaluation of interventions to improve PCMC.

## Methods

Here we discuss the scale development and validation process used in this study, which took place in both Kenya and India. We focus on the Kenyan data in this paper and where relevant, the components of the process that took place in India are mentioned. The results of the development and validation process in India will be presented in a separate analysis. We used the following standard procedures for scale development and validation [45, 46].

### Defining the construct of person-centered maternity care and identifying domains

As PCMC is a relatively new concept in developing settings, we examined bodies of work that discuss overlapping issues related to PCMC, though do not necessarily use terms such as PCMC. This includes literature from health system responsiveness [47–49], perceived quality of care [50, 51], mistreatment of women during childbirth [13, 14, 22], and the general literature on quality of care for maternal health [24, 28, 52–54]. In addition, we examined the general literature on person-centered care, which is mostly from developed settings [55–58]. Although framed differently, these separate bodies of work include important aspects of PCMC.

Following this review, we adopted the following definition of person-centered maternity care: “Providing maternity care that is respectful and responsive to individual women and their families’ preferences, needs, and values, and ensuring that their values guide all clinical decisions,” a definition from the Institute of Medicine [57]. PCMC includes timely and equitable care. We identified 10 domains of PCMC, namely:

1. Dignity and Respect
2. Autonomy
3. Privacy and Confidentiality
4. Communication
5. Social Support
6. Supportive Care
7. Predictability and Transparency of Payments
8. Trust
9. Stigma and Discrimination
10. Health Facility environment

### Item generation

Following the identification of these domains, we developed an item pool with questions capturing each of the domains. Many of the questions were based on questions used in existing tools addressing one or more of the domains of interest [13, 44, 49–51]. The first draft of our tool contained approximately 40 items, which were statements with 5-point response options ranging from 1: “strongly agree” to 5: “strongly disagree”.

### Expert reviews

The domains and items were then evaluated through expert reviews. Our internal team initially reviewed the items individually and in several group discussions. We then sent revised versions to other maternal health academic experts to review. We received individual inputs from six maternal health experts outside our core team. A formal expert review was then conducted by bringing together eight Maternal and Child Health experts in Kenya to review the items in a focus group discussion format. These experts included academic researchers, as well as public health and clinical practitioners with several years of experience. The meeting was held in a conference room at the Kenya Medical Research Institute. The expert reviews yielded suggestions for rewording many questions, as well as inclusion of additional questions for some of the domains. Following expert review, the number of items had increased to approximately 70 questions, and included multiple ways of asking the same questions. Some expert reviewers also strongly recommended against using the “strongly agree” to “strongly disagree” response format, which has been shown to have high acquiescence bias [59]. Thus, we framed the questions in two ways for testing following expert reviews. An initial set had the statements with response options in the form of “strongly disagree,” “disagree,” “neither disagree nor agree,” “agree,” or “strongly agree;” while the secondary set contained questions with frequency responses in the form of “never”, “a few times”, “sometimes”, “most of the time”, and “all the time”.

### Cognitive interviews

Cognitive interviews are an integral component of scale construction [60]. Cognitive interviews were conducted to improve our understanding of how participants internalized the questions; assess if the questions were being interpreted as intended; evaluate problems with the wording of questions; evaluate whether questions were context appropriate and salient; and finally, to assess appropriate length of the tool [60–62].

The initial cognitive interviews for this project were conducted in India. Six cognitive interviews were conducted with women post-delivery in two government facilities in Uttar Pradesh in March, 2016. Interviews were conducted by two teams of two interviewers each, with one acting as a note taker. Respondents were eligible if they were between 18 and 49 years, had just delivered in the postpartum ward, had not had a cesarean section, and felt well enough to be interviewed. Recruitment and informed consent took place in the labor ward. Interviews were conducted in Hindi and detailed notes were taken by one of the research team members and then translated into English.

Through review of the initial Indian interviews, the research team learned that respondents struggled when provided with statements and asked to state if they “strongly disagreed,” “disagreed,” “neither disagreed nor agreed,” “agreed”, or “strongly agreed”; a proportion of the respondents simply replied “sometimes” to a majority of the questions. Thus, all questions were changed to utilize the frequency format as suggested by our expert reviewers in Kenya. In addition, the middle response option (“sometimes”) was dropped, as the majority of respondents frequently gravitated towards this response. While translating interviews into Swahili and Luo, the research team learned that the distinction between a “few times” and “sometimes” was not clear in the translated versions, further supporting the need to drop the middle category. Questions were revised following the initial cognitive interviews to a set of approximately 60 questions, and a subsequent round of cognitive interviews were conducted with women in Kenya.

The cognitive interviews in Kenya were conducted between May and June 2016 at three government health facilities in Kiambu County, by three female interviewers trained in cognitive interviewing. Working closely with facility staff, interviewers purposively identified ten women for the interviews. Respondents were eligible if they were aged 18-49 years, delivered in the preceding 7 days at one of the study facilities, and felt well enough to participate. Recruitment and written informed consent took place in a private space within facility grounds to ensure confidentiality. Respondents were also asked if the interview could be audio-recorded during the consenting process, though this did not constitute an eligibility criterion. At the time of consent, respondents were given the option of continuing with the cognitive interview in a private space at the facility or having the interview conducted at their home within the next few days. All but one of the interviews occurred at a private space in the health facility, and all gave consent for audio recording. Cognitive interviews were conducted in English and/or Swahili based on the respondent’s language preference. Mobile phone airtime credit in the amount of approximately $1.50 was provided to respondents to thank them for their participation in the study. Audio recordings were transcribed verbatim and concurrently translated to English, if necessary, by independent consultants. Quality assurance checks were performed on all transcripts by comparing them to the audio recordings.

A cognitive interview guide developed by the research team directed interviewers to ask how frequently a person-centered care indicator occurred, followed by a rating of the importance of the indicator, with probes to understand why/why not, and/or in which circumstance each item would be appropriate/inappropriate (e.g. being called by name, being shouted at or scolded by a provider, etc.).

Respondents were also asked if they found the questions difficult to understand, and if so, how they thought the question could be improved. Probes included: How did you arrive at that answer? Was this question difficult for you to answer? How would you rephrase this question to make it better? When a question was framed in multiple ways, respondents were asked which of the questions they preferred with regard to ease of understanding.

The research team then examined the distribution of responses as well as the ratings of their importance. Transcripts were reviewed to identify ambiguous or confusing questions, and responses to as to why respondents answered the way they did. Following these analyses, questions that did not work well in the cognitive interviews were removed and those that seemed unclear revised. This exercise reduced the number of items to 38, with each question containing a 4-point response scale: “no, never”, “yes, a few times”, “yes, most of the time” and “yes, all the time.” In addition, responses for two questions on verbal and physical abuse were changed to (“no never”, “yes, once,” “yes, a few times”, and “yes, many times”) to account for the low prevalence of overt abuse, while retaining the same scale as the other responses options. A “not applicable” response option was added to questions where the cognitive interviews revealed that the question might not be relevant to all respondents. Revised items were then pretested with the full questionnaire among a convenience sample of about 39 women in the participating facilities. Final revisions were then made, although minor at this point in the refinement process.

### Translation

The translation of the tool was an iterative process, starting before the cognitive interviews and continuing until the version used in the survey was finalized. We recognized that nuances in language could affect the meaning of the questions, and some of the words in the English version may not have words in the local languages that directly translated to how they were used in English. To handle this, we spent a substantial amount of time during training of field officers to ensure that questions had the same meaning, even if the words used were a bit more colloquial. The tool was first translated into Swahili by someone who could speak both English and Swahili. During training of the field officers for the cognitive interviews, additional changes were made to the translated versions based on input from the field officers who spoke both English and Swahili. An example is the use the slang phrase, “kitu kidogo”, directly translating to *something small*, instead of bribe which we originally used. Field officers suggested we use this phrase for bribe as this is how it was best understood by Kenyans. The Luo translation went through a similar process of discussing the questions with the field officers during their training for the surveys. The final translated versions were based on consensus with the field team. Given the group input of multiple local language speakers to the tool, we believe the questions in the different languages were similar in meaning.

### Survey

The final set of items was administered as part of two separate surveys in Kenya: in a rural setting and an urban setting.

#### Rural sample

In Migori County, a predominantly rural county in western Kenya, a survey was conducted in August and September 2016. The sample was comprised of women who delivered in the 9 weeks preceding the survey in the County. Women were recruited at health facilities (in the delivery wards and postnatal clinics), and in their homes. A multistage sampling approach was used to select women. First, the county was divided into 8 strata based on the 8 sub-counties in the county. All health units in each stratum were then identified and10 health units randomly selected. Within each selected health unit, women who delivered in the preceding 9 weeks were identified with the help of the Community Health Volunteer assigned to that health unit. The target was to conduct approximately 200 interviews in each sub-county. The first 20 eligible women in each health unit who were available were interviewed. If the target was not met after interviews in all the selected health units were completed, more health units in that sub-county were sampled. Twelve trained data collectors conducted the interviews, with one interviewer from each sub-county and an additional interviewer in the four larger sub-counties. The interviews were conducted in English, Swahili, and Luo in private spaces in health facilities or in the homes of the respondents. All participants provided written informed consent after receiving information about the research. They were given a gift of 200 Kenyan shillings (~$2). The majority of data was collected using the RedCap application, with data uploaded directly online. In instances where the Internet connection was poor, the interviews were entered on paper and transferred to RedCap when the data collector reached a place with better connectivity. Quality assurance checks were performed throughout the data collection. A total of 1052 women were interviewed, with a response rate above 98%. We performed psychometric analysis using data from women who delivered in a health facility (877) and who had complete information on all the items (*N* = 857).

#### Urban sample

A second survey was conducted from August through December 2016 at seven government health facilities in Nairobi and Kiambu Counties using the same PCMC tool. Nairobi is the National capital of Kenya and is 100% urban. Kiambu County is 60% urban, but our sample was drawn from the urban portions of the county [63]. The sample was comprised of women who delivered within a week of the survey in any of the seven participating health facilities. The post-partum length is shorter here because this was the target group for this project, and we did not have the flexibility of changing this sample. Six trained interviewers conducted the interviews. Respondents were identified with the help of health of facility staff and invited to participate in the survey. Recruitment and consenting took place in a private space within facility grounds, and respondents were given the option of continuing with the interview in a private space at the facility or having the interview at their home within the next few days. All but three of the interviews occurred at a private space in the health facility. Interviews were conducted in English and/or Swahili. All participants provided written informed consent after receiving information about the research. Respondents were given mobile phone airtime worth approximately $1.50 in appreciation of their participation. Interviews were conducted using the SurveyCTO platform, with data uploaded to the server at the end of each day. Quality assurance checks were performed throughout the data collection. A total of 531 women were interviewed. We performed the psychometric analysis using data from women who had complete information on all the items (*N* = 530).

### Psychometric analyses

We first examined the distributions of all the items, comparing the two samples. In instances where questions had responses in the “not applicable” category, we decided to convert the “not applicable” category into the highest category to obtain a uniform scale for the psychometric analysis. This approach is conservative as it assumes the highest quality rating for each “not applicable” response. For example, for the question on labor support, we assume that someone who said “they did not want a support person” would have been allowed one if they so desired. We reverse coded negative items in order for responses to reflect a scale of 0 as the lowest level to 3 as the highest level. We then constructed a correlation matrix to examine the correlations among the items.

We conducted the psychometric analysis to assess the validity and reliability of the tool. Validity is the degree to which the items in a survey tool measure the phenomenon or construct it is intended to measure [64]. Common types of validity that need to be considered in scale development are content, construct, and criterion related validity [46]. Content validity assesses whether the items represent all possible indicators relevant to the construct [46]. We assured content validity through a comprehensive literature search to develop a definition for the construct, to identify related domains based on empirical research and theory, and then developing items that represent each domain identified. The expert reviews were also used to optimize content validity.

Construct validity is the degree to which a measure relates to other measures in theoretically predictable ways, or how well the items represent the underlying conceptual structure [46, 64]. Factor analysis is an important step in psychometric analysis. It is used to examine the interrelationships among a set of variables, thus, can be used to assess construct validity. Factor analysis is also a data reduction method used to re-express data on multiple variables with fewer dimensions and to reduce a set of observed variables to a smaller, more parsimonious set of variables [45, 46, 65]. We conducted exploratory factor analysis using principal factoring. We used the Kaiser-Meyer-Olkin (KMO) measure of sampling adequacy to assess if the variables were suitable for factor analysis. The KMO measure has values between 0 and 1, with small values indicating that overall, the variables have little in common to warrant a principal components analysis. Values above 0.5 are considered satisfactory for factor analysis [66]. We used a KMO value of 0.5 as the criterion for sampling adequacy.

The factor analysis was an iterative process. First, we conducted the factor analysis for the rural and urban samples separately, and also with the combined sample. We then examined the Eigenvalues (the amount of information captured by a factor) and scree plots (plots of Eigenvalues) to determine the number of factors to extract. We used both Kaiser’s rule of retaining only factors with eigenvalues exceeding unity and the “break” in the scree plot to decide on how many factors to retain [45, 46, 65]. We then conducted subsequent factor analysis and examined the item loadings to determine which items to retain or delete. Item loading is the degrees to which the original item scores correlate with the components. We used a cut off of 0.3 at the initial stage [67]. Items that did not have a loading of 0.3 or higher on any of the extracted factors in the 3 samples were thus dropped after the first set of factor analysis. Further rounds of factor analysis were conducted to decide on the final set of items and sub-scales. In these subsequent stages, the cut off for deletion was varied based on the theoretical importance of the item.

Factor rotations are used to simplify the interoperability of factor solutions and to facilitate the interpretation of the results [66]. Orthogonal rotation preserves the perpendicularity of the rotated components and assumes the factors are uncorrelated. Oblique rotation, however, allows for correlation between the rotated factors and aligns the factor axes as closely as possible to the groups of the original variables [45, 60, 66]. As the PCMC domains were theoretically related and the extracted components were correlated, we used oblique rotation. We tested our final factor structure in confirmatory factor analysis with various samples stratified by setting, location of interview, postpartum length, age of respondent, and educational level of respondent. We also examined the Pearson correlation coefficient between the components identified by factor analysis to assess construct validity.

Criterion-related validity refers to whether the measure is related to other measures or outcomes in theoretically predictable ways [46, 64]. One approach to determining criterion validity is through hypothesis testing [67]. Consistent with other work we hypothesized that the PCMC scale would be correlated with global measures of satisfaction with care and quality of care [28, 44]. We tested this by regressing the main scale and sub-scales on women’s ratings of their satisfaction with the services, the quality of care they received during delivery, and whether she would deliver in the same facility if she were to have another baby.

Reliability refers to the degree to which a measurement tool produces stable and consistent results [46]. For a measure to be valid, it must also be reliable, but a reliable measure may not necessarily be valid [64]. We assessed the internal consistency reliability using Cronbach’s alpha. Cronbach’s alpha ranges from 0 to 1. Higher score imply greater reliability; with 0.7 or higher generally considered sufficient evidence of reliability [45]. An extremely high alpha (>0.95) might however suggest redundancy among some indicators [46]. Reliability across settings was also examined by testing for the difference between scores in the rural and urban sample. We used STATA version 14 to perform the statistical analyses.

## Results

Table 1 shows the demographic characteristics of respondents for the urban and rural samples. The average age is about 25 years for the rural sample, and 26 years for the urban sample. Approximately 79% of the women in the rural sample are married, compared to 72% for the urban sample. Women in the urban sample are slightly more educated than those in the rural sample. Close to 40% of the interviews in the rural sample occurred at a health facility, as compared to all the interviews in the urban sample. The postpartum length for women interviewed in the rural sample is distributed between zero and 9 weeks. Only women less than a week postpartum were interviewed in the urban sample.

**Table 1:** Distribution of selected demographic variables

	Rural		Urban		Total	
	No.	%	No.	%	No.	%
Age: Mean (SD)	857	25.0 (5.9)	530	25.6 (4.8)	1387	25.2 (5.5)
Parity: Mean (SD)	856	2.8 (2.0)	530	2.1 (1.1)	1386	2.5 (1.7)
Marital status						
Single	136	16	61	12	197	14
Partnered/Cohabiting	3	0	75	14	78	6
Married	676	79	382	72	1058	76
Widowed	32	4	1	0	33	2
Divorced/Separated	10	1	11	2	21	2
Highest grade completed						
Primary or less	483	56	204	39	687	50
Post-primary/Vocational/Secondary	265	31	241	46	506	37
College or above	109	13	85	16	194	14
Literacy: reading						
No, cannot read	36	4	2	0	38	3
Yes, but with some difficulty	127	15	33	6	160	12
Yes, Very well	694	81	495	93	1189	86
Literacy: writing						
No, cannot write	30	4	3	1	33	2
Yes, but with some difficulty	140	16	29	6	169	12
Yes, Very well	687	80	498	94	1185	85
Interview language ^a^						
English	108	13	257	48	365	26
Swahili	254	30	274	52	529	38
Luo	495	58			495	36
Total	857	100	530	100	1387	100

^a^ For the urban sample this is the language the consent was completed in. The interview language was not specified

Table 2 shows the original domains, the questions for each domain, and comments on decisions taken related to that item. The distributions for the items are shown in Additional file 1: Appendix 1. With few exceptions, the responses generally ranged between 0 and 3. Notably, the responses for the labor and delivery support questions in the urban sample had a large proportion of responses in the “not applicable” category. Over 40% of respondents in the urban sample did not want a support person during labor or delivery. This category was recoded into the “all the time” category for the psychometric analysis and likely has implications for the factor structure obtained for the urban sample.

**Table 2:** Items for person-centered maternity care scale

Original Domain	Question	Referred to in text as	Comment
Dignity/Respect	1. How did you feel about the amount of time you waited? Would you say it was very short, just a little long, somewhat long, or very long?	Time to care	Retained
Dignity/Respect	2. During your time in the health facility did the doctors, nurses, or other health care providers introduce themselves to you when they first came to see you?	Introduce self	Retained but loads better in rural than urban sample
Dignity/Respect	3. Did the doctors, nurses, or other health care providers call you by your name?	Called by name	Retained but loads better in rural than urban sample
Dignity/Respect	4. Did the doctors, nurses, or other staff at the facility treat you with respect?	Treated with respect	Retained
Dignity/Respect	5. Did the doctors, nurses, and other staff at the facility treat you in a friendly manner?	Friendly	Retained
Dignity/Respect	6. Did the doctors, nurses, and other staff at the facility show they cared for you?	Show cared	Deleted: correlated with friendly and respect
Dignity/Respect	7. Did you feel the doctors, nurses, or other health providers shouted at you, scolded, insulted, threatened, or talked to you rudely?	Verbal abuse	Retained
Dignity/Respect	8. Did you feel like you were treated roughly like pushed, beaten, slapped, pinched, physically restrained, or gagged?	Physical abuse	Retained but loads better in rural than urban sample
Dignity/Respect	9. Did you feel like you were forced to stay at the health facility against your will because you could not pay your bill?	Stay against will	Deleted: low correlation with other items and low loading in all samples
Privacy/Confidentiality	10. When you were speaking to the doctors, nurses or other staff at the facility, did you feel other people not involved in your care could hear what you were discussing?	Auditory privacy	Deleted: low correlation with other items and low loading in all samples
Privacy/Confidentiality	11. During examinations in the labor room, were you covered up with a cloth or blanket or screened with a curtain so that you did not feel exposed?	Visual privacy	Retained
Privacy/Confidentiality	12. Do you feel like your health information was or will be kept confidential at this facility?	Record confidentiality	Retained
Autonomy	13. Did you feel like the doctors, nurses or other staff at the facility involved you in decisions about your care?	Involvement in care	Retained
Autonomy	14. Did the doctors, nurses or other staff at the facility ask your permission/consent before doing procedures and examinations on you?	Consent to procedures/exams	Retained
Autonomy	15. During the delivery, do you feel like you were able to be in the position of your choice?	Delivery position choice	Retained but loads better in urban than rural sample
Communication	16. Did the doctors, nurses or other staff at the facility speak to you in a language you could understand?	Language	Retained
Communication	17. Did the doctors and nurses explain to you why they were doing examinations or procedures on you?	Explain exams/procedures	Retained
Communication	18. Did the doctors and nurses explain to you why they were giving you any medicine?	Explain medicines	Retained
Communication	19. Did you feel you could ask the doctors, nurses or other staff at the facility any questions you had?	Able to ask questions	Retained
Social Support	20. Were you allowed to have someone you wanted (outside of staff at the facility, such as family or friends) to stay with you during labor?	Labor support	Retained
Social Support	21. Were you allowed to have someone you wanted to stay with you during delivery?	Delivery support	Retained but loads better in urban than rural sample
Supportive Care	22. Did the doctors and nurses at the facility talk to you about how you were feeling?	Talk about feeling	Retained
Supportive Care	23. Did the doctors, nurses or other staff at the facility support your anxieties and fears?	Support anxiety	Retained
Supportive Care	24. Did the doctors and nurses ask how much pain you were in?	Ask about pain	Deleted: correlated with control pain and ask about feeling
Supportive Care	25. Do you feel the doctors or nurses did everything they could to help control your pain?	Control pain	Retained
Supportive Care	26. When you needed help, did you feel the doctors, nurses or other staff at the facility paid attention?	Attention when need help	Retained
Supportive Care	27. Did you feel the doctors and nurses paid attention to you during your stay in the facility?	Attention during stay	Deleted: correlated with attention when needing help
Supportive Care	28. Were you allowed to eat or drink when you were hungry/thirsty?	Allowed to eat/drink	Deleted: low correlation with other items and low loading in all samples
Trust	29. Did you feel the doctors, nurses or other staff at the facility took the best care of you?	Took best care	Retained
Trust	30. Did you feel you could completely trust the doctors, nurses or other staff at the facility with regards to your care?	Trust	Retained
Predictability &transparency of payments	31. During your time at the facility, did any staff at the facility ask you or your family for kitu kidogo? (*colloquial translation for bribe)*	Bribe	Deleted: low correlation with other items and low loading in all samples
Stigma & Discrimination	32. During your time in the health facility, would you say you were treated differently because of any personal attribute… like your age, marital status, number of children, your education, wealth, your connections with the facility, or something like that?	Differential treatment	Deleted: low correlation with other items and low loading in all samples
Facility environment	33. Do you think there was enough health staff in the facility to care for you?	Enough staff	Retained
Facility environment	34. Thinking about the labor and postnatal wards, Did you feel the health facility was crowded?	Crowded	Retained but loads better in urban than rural sample
Facility environment	35. Thinking about the wards, washrooms and the general environment of the health facility, will you say the facility was very clean, clean, dirty, or very dirty?	Clean	Retained but loads better in rural than urban sample
Facility environment	36. Was there water in the facility?	Water	Retained
Facility environment	37. Was there electricity in the facility?	Electricity	Retained
Facility environment	38. In general, did you feel safe in the health facility?	Safe	Retained

**Fig. 1:**
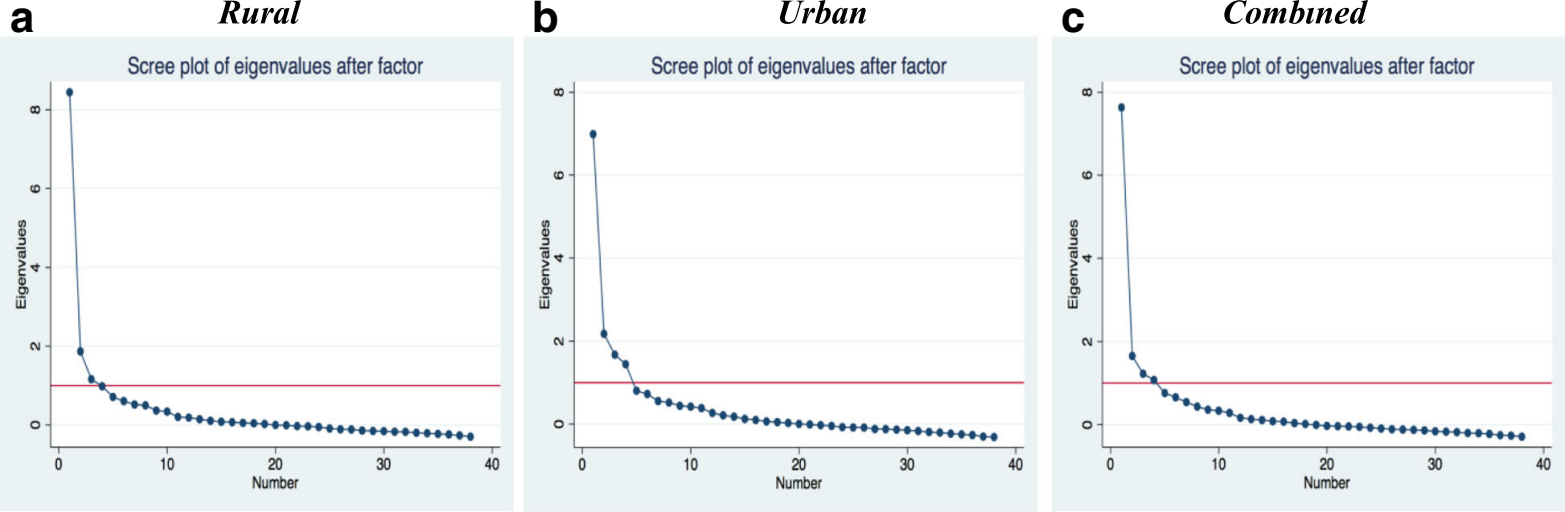
Scree plot of eigenvalues after factor analysis for the rural, urban, and combined samples

There is good correlation among the majority of items, with correlations between 0.2 and 0.8. None of the items had correlations >0.8. Five items had correlations of <0.2 with all other items. The KMO measure of sampling adequacy for all items are greater than 0.5, with an overall KMO of 0.91, indicating that overall the variables are satisfactory for factor analysis. The initial exploratory factor analysis yielded 3 factors with eigenvalues of greater than one for the rural sample, accounting for 84% of the variance among the items. For the urban sample, the exploratory factor analysis yielded four factors with eigenvalues of greater than one accounting for 86% of the variance. When the two samples were combined, we had four factors with eigenvalues of greater than one, accounting for 91% of the variance. When we examine the difference in eigenvalues between the factors and scree plots (Fig. 1a, b and c) for each sample, we find that even though there are three or four factors with eigenvalues of more than one, there is one dominant factor in all samples. This means that depending on which criteria we use for factor extraction, we could have three or four sub-scales or just one unified scale.

In the next stage of the analysis we retained only items that had factor loadings of greater than 0.3 on the factors with eigenvalues >1 (in any of the 3 samples). We dropped 5 items (see Table 2) based on this criterion. These items also had low correlations with the rest of the items in the correlation matrix. To further reduce the number of items, we again examined the correlations among the remaining items to identify items that were conceptually very similar (but only reasonably correlated (i.e. *r* > 0.6) and so not excluded based purely on their correlation). Three more items were dropped, with a decision made on which of the correlated items to drop based on theoretical considerations. For example, the item on whether providers “showed they cared” was correlated with both “treated with respect” and “treated in a friendly manner,” thus was dropped. “Being asked about pain” was correlated with “control of pain” and “being asked about feelings”, thus “being asked about pain” was dropped and “control of pain” and “being asked about feelings” were retained, as these items were more encompassing. Additionally, “paid attention during stay” and “paid attention when they needed help” were correlated; therefore “paid attention during stay” was deleted, as it was less specific. Items deleted and the rationales for deletion are shown in Table 2. Thirty items remained after this process, including items from all of the original domains, with the exception of discrimination and transparency and predictability of payments.

Another round of factor analysis with the 30 items yielded two factors with eigenvalues greater than 1 for the rural sample and four factors for the urban sample. When the point of the “break” in the scree plot was used as the criteria for factor extraction, it suggested one single underlying factor structure for both samples. Thus, we ran another set of factor analysis retaining only one factor for all the samples with the remaining 30 items. Loadings of the items onto this factor were each >0.2, with the exception of the following: “Physical abuse” loaded at less than 0.2 on both samples; “position of choice”, “delivery support”, and “crowding” loaded at less than 0.2 in the rural sample; and “provider introduce self” and “called by name” loaded at less than 0.2 in the urban sample (shown on Table 3). In the combined sample, the items with low loadings on the single factor were “provider introduce self”, “physical abuse”, “position of choice”, “delivery support”, and “crowding.” This suggests that one unified person-centered maternity care scale could be constructed, with between 24 and 30 items, depending on whether we used a purely statistical criterion and dropped all items with low loadings or included with low loadings based on theoretical relevance.

**Table 3:** Rotated factor loadings of items on dominant factor for main scale

	Rural	Urban	Combined
Variable	Rotated factor loading		
1. Time to care	0.39	0.26	0.32
2. Introduce self	0.21	0.12	0.19
3. Called by name	0.59	0.17	0.44
4. Treated with respect	0.70	0.67	0.69
5. Friendly	0.65	0.68	0.66
6. Verbal abuse	0.18	0.39	0.26
7. Physical abuse	0.11	0.06	0.10
8. Visual privacy	0.49	0.36	0.43
9. Record confidentiality	0.53	0.52	0.52
10. Involvement in care	0.59	0.44	0.52
11. Consent to procedures/exams	0.61	0.49	0.56
12. Delivery position choice	0.06	0.35	0.15
13. Language	0.46	0.35	0.42
14. Explain exams/procedures	0.66	0.53	0.61
15. Explain medicines	0.49	0.34	0.43
16. Able to ask questions	0.58	0.48	0.54
17. Labor support	0.33	0.35	0.31
18. Delivery support	0.05	0.34	0.14
19. Talk about feeling	0.59	0.47	0.55
20. Support anxiety	0.45	0.30	0.39
21. Attention when need help	0.66	0.64	0.65
22. Took best care	0.67	0.66	0.67
23. Control pain	0.38	0.40	0.39
24. Trust	0.65	0.63	0.64
25. Clean	0.25	0.36	0.28
26. Safe	0.55	0.59	0.56
27. Enough staff	0.55	0.42	0.51
28. Crowded	0.06	0.21	0.11
29. Water	0.45	0.28	0.39
30. Electricity	0.40	0.32	0.36

On the other hand, if we used Kaiser’s rule of retaining factors with eigenvalues exceeding unity, then we would have had between 2 and 4 factors (or sub-scales) making up our PCMC scale. To assess this, we conducted another set of factor analysis with the 30 items, retaining 3 factors for each sample. In the rural sample, all items except those on verbal and physical abuse load onto to the first two factors, with the more subjective measures (e.g. “treated with respect”, “treated in friendly manner”) tending to load on the first factor and the less subjective (e.g. “providers introduce themselves”, “called by name”) loading on the second factor. Only verbal and physical abuse loaded on the third factor, although verbal abuse also had a reasonably high loading (EV = 0.25) with other items on dignity and respect on the first factor. The items on health facility environment also loaded onto the first factor, except for “crowding,” which had low factor loading (less than 0.1) on all 3 retained factors in the rural sample. Most of the items on communication loaded on the second factor.

For the urban sample, however, only “labor and delivery support” and “position of choice” questions loaded onto the third factor, with most of the communication and autonomy related items loading on first factor and the rest on the second factor. Verbal abuse loaded on the first factor with the other items on dignity and respect, and physical abuse did not load on any of the retained factors in the urban sample. “Crowding” loaded with the other health facility environment items in the urban sample. A few items also loaded on more than one factor. We decided not to use cross loading as a sole criterion for item deletion at this stage. Instead, the distribution of the items, theoretical rationale, and the judgment of the study team was used [44]. If an item cross-loaded on more than one factor, the item was retained in the factor it loaded highest on. The difference in factor loadings was greater than 0.1 in most cases, which was judged sufficient to warrant this approach.

The factor analysis thus suggested a unified scale with possibility of 3 sub-scales based on the factors extracted. However, because the factors extracted included a mix of items from each of the original domains, it was difficult to ascertain what each factor represented conceptually. We therefore decided to regroup the retained items into 3 sub-scales based on the factor loadings and conceptual domains drawn from the experience of care categories in the WHO quality of care framework for maternal and newborn care. We created sub-scales for: Dignity and Respect (DR), Communication and Autonomy (CA), and Supportive Care (SC). We then conducted factor analysis with the items in each of these groups, in an iterative process, moving items that did not load in their assigned group until each loaded well with a group. We considered having a separate sub-scale for the items related to health facility environment (HFE), as they seemed conceptually distinct from the other items focused on interpersonal interactions. But we decided against a separate HFE sub-scale because it had low reliability as a subscale. We decided to retain these items in the SC sub-scale because they are needed to provide supportive care, and most of them loaded well with other items in that group. Each sub-scale yielded one factor, with most items loading reasonable well onto the extracted factor.

As shown in Table 4, the factor loadings were at least 0.2, with most greater than 0.40. The exceptions were “physical abuse,” “called by name,” “delivery support,” “crowding,” and “cleanliness,” which had loadings of less than 0.2 on their sub-scales. Of note, “cleanliness” had a negative loading on the SC sub-scale in the urban sample. We considered cleanliness might be more representative of dignity and respect, thus, we run the factor analysis retaining this item in the DR sub-scale. But it loaded negatively on the DR sub-scale for the urban sample. Factor loadings for cleanliness on the SC sub-scale were slightly higher than that on DR sub-scale in the rural and combined samples. Furthermore, with the confirmatory factor analysis utilizing the combined sample, the coefficient for cleanliness was significant (*p* < 0.05) in the SC sub-scale, but not in the DR sub-scale. Therefore, we maintained cleanliness in the SC sub-scale. The coefficients for all the other items were significant in the confirmatory factor analysis.

**Table 4:** Rotated factor loadings on dominant factor for sub-scales

		Rural	Urban	Combined
Sub-scale	Item	Rotated factor loading		
Dignity and respect				
	Treated with respect	0.79	0.77	0.78
	Friendly	0.79	0.80	0.80
	Verbal abuse	0.34	0.44	0.39
	Physical abuse	0.22	0.10	0.18
	Visual privacy	0.42	0.30	0.36
	Record confidentiality	0.47	0.48	0.45
Communication and autonomy				
	Introduce self	0.24	0.20	0.23
	Called by name	0.59	0.17	0.43
	Involvement in care	0.66	0.39	0.58
	Consent to procedures	0.75	0.61	0.67
	Delivery position choice	0.13	0.38	0.22
	Language	0.37	0.32	0.36
	Explain exams/ procedures	0.77	0.67	0.73
	Explain medicines	0.57	0.42	0.51
	Able to ask questions	0.59	0.40	0.53
Supportive Care				
	Time to care	0.38	0.28	0.31
	Labor support	0.28	0.43	0.29
	Delivery support	0.01	0.42	0.12
	Talk about feeling	0.54	0.41	0.50
	Support anxiety	0.40	0.32	0.35
	Attention when need help	0.67	0.61	0.65
	Took best care	0.74	0.68	0.73
	Control pain	0.39	0.35	0.39
	Trust	0.72	0.66	0.70
	Enough staff	0.59	0.44	0.53
	Crowded	0.05	0.26	0.12
	Clean	0.27	−0.36	0.07
	Water	0.51	0.32	0.45
	Electricity	0.43	0.30	0.38
	Safe	0.62	0.62	0.63

The factor analysis using the full sample, as well as for samples stratified by setting, place of interview, postpartum length, age, and education, yielded similar results (results not shown) with “physical abuse,” “delivery position choice,” “delivery support person,” “crowding,” and “cleanliness” being the only items that did not consistently have factor loadings of >0.2 in all the samples. Therefore, as with the main scale, if we were to decide on the scale based purely on the statistical analysis, these 5 items would be dropped to have 25-items that work reasonably well in both rural and urban settings. However, given the theoretical significance of these items and that some loaded relatively well in one sample but not the other, we have decided to retain them in the current version of the scale to be tested in future validation studies. The sub-scales are strongly correlated with each other, with correlation coefficients (r) ranging from 0.53 to 0.63, and with the main scale (*r* = 0.75, 0.86, and 0.9 for DR, CA, and SC respectively).

The full 30-item PCMC scale has good internal consistency reliability, with Cronbach’s alpha of 0.88 for the rural sample, 0.83 for the urban sample, and 0.86 for the combined sample. Dropping “physical abuse,” “delivery position choice,” “delivery support person,” and “crowding” only marginally increased the alphas to 0.89 and 0.87 for the rural and combined samples respectively. The alpha for the urban sample does not change due to dropping these items. The Cronbach’s alphas for the sub-scales for Dignity and Respect, Communication and Autonomy, and Supportive Care for both the rural sample and urban samples are within acceptable ranges from 0.6 to 0.8 (Table 5). Dropping “physical abuse” marginally increases the alpha for the DR sub-scale to 0.67 for the rural sample and to 0.64 for the urban scale. Dropping “delivery position choice” increases the alpha for CA sub-scale for the rural sample to 0.80 and marginally decreases that for the urban sample to 0.61. Dropping “delivery support,” “crowding,” and “cleanliness” increases the alpha for SC sub-scale for the rural sample to 0.79 and decreases that for the urban sample to 0.69. Thus, improving reliability is not a compelling reason for dropping these items.

**Table 5:** Reliability and distribution of Full PCMC scale and sub-scales

	Alpha	Mean	SD	Min	Max
Rural					
Full PCMC Scale	0.88	59.5	13.6	21.0	90.0
Dignity and respect	0.66	15.1	2.9	3.0	18.0
Communication and autonomy	0.78	13.9	5.9	1.0	27.0
Supportive Care	0.75	30.5	6.8	8.0	45.0
Urban					
Full PCMC Scale	0.83	60.2	12.3	22.0	86.0
Dignity and respect	0.61	14.4	2.9	3.0	18.0
Communication and autonomy	0.62	15.1	4.7	3.0	26.0
Supportive Care	0.72	30.4	6.5	10.0	44.0
Combined					
Full PCMC Scale	0.86	59.8	13.1	21.0	90.0
Dignity and respect	0.63	14.8	2.9	3.0	18.0
Communication and autonomy	0.73	14.4	5.5	1.0	27.0
Supportive Care	0.72	30.5	6.7	8.0	45.0

The mean PCMC score (based on the sum of all the items in the scale) for the rural sample is 59.5 (SD = 13.6) with a range of 21 to 90, and that for the urban sample is 60.2 (SD = 12.3), with a range of 22 to 86. The difference is not significant (*p* = 0.85). The means for the sub-scales are also shown in Table 5. The differences between the means for the rural and urban samples are significant (*p* < 0.001) for DR and CA, although not for SC.

The regression of each of the sub-scales and the full scale on patients’ ratings of satisfaction with services, general quality ratings, and whether the woman would deliver in the same facility if she were to have another baby shows the sub-scales are individually and collectively correlated with the global measures of satisfaction and quality of care, which suggests high criterion validity. Table 6 shows the bivariate linear regressions for these global measures on the PCMC scale (reversed for ease of interpretation and to show graded increase in global measures with increasing PCMC scores).

**Table 6:** Bivariate linear regression of person-centered maternity care score on global measures of satisfaction with maternity services

	Coef.	*P*-value	95% Conf.	Interval
Level of Satisfaction				
Dissatisfied (ref)				
Neither satisfied nor dissatisfied	0.66	0.80	−4.33	5.66
Satisfied	10.83	0.00	6.84	14.82
Very satisfied	17.44	0.00	13.28	21.60
Constant	48.26	0.00	44.34	52.17
Rating of quality of care				
Poor (ref)				
Fair	3.00	0.28	−2.48	8.47
Good	12.67	0.00	7.76	17.57
Very good	16.46	0.00	11.47	21.46
Excellent	20.64	0.00	14.37	26.91
Constant	46.73	0.00	41.90	51.56
Will deliver in same place again				
No (ref)				
Yes, somewhat	0.07	0.97	−3.77	3.92
Yes, definitely	7.64	0.00	4.24	11.04
Constant	53.84	0.00	50.60	57.08

## Discussion

The World Health Organization includes women’s experiences of care and person-centered outcomes as primary components in their quality of care framework for maternal and newborn health [24]. There is however no consensus on how to measure these constructs. We describe the process of developing and validating a scale to measure person-centered maternity care. We present a 30-item scale that can be used to measure women’s perceptions of person-centered care during labor and delivery, and show that it is has high validity and reliability in both rural and urban settings in Kenya. The scale has high content validity based on our extensive literature and expert reviews. The exploratory factor analysis suggests high construct validity—the items measure an underlying construct, which we believe to be PCMC based on the content validity. It also has high criterion validity, being strongly correlated with global measures of satisfaction and quality of maternity care. In addition, it has high internal reliability, with an alpha well above the recommended level of 0.7. There currently is no gold-standard tool in this area of work, hence we are unable to test the performance of the PCMC tool against a gold standard. We present the 30-item scale with three sub-scales for “Dignity and respect,” “Communication and autonomy,” and “Supportive care.” These sub-scales also have good content, construct, and criterion validity, with reliability within acceptable ranges of 0.6 to 0.8.

We used DeVellis’ guidelines in scale development, which include use of theory, specificity of measures, and choosing items that reflect the purpose of the scale to guide items to include [46, 68]. As our goal was to develop a theory based but practical PCMC scale that can be easily administered in various contexts, we decided to include five items that differed in factor loadings across urban and rural contexts to be conservative and over inclusive, rather than over exclusive. Redundancy is recommended in early stages of scale development to achieve inclusiveness [68]. We retained “physical abuse” it has been shown to be important to PCMC in extant literature and is common in many global contexts [13, 14, 22]. Similarly, we retained the items on “delivery support,” “delivery position choice,” “crowding,” and “cleanliness,” as these are salient aspects of PCMC [22, 28, 34, 69–71]. Such inclusiveness is necessary to construct a measure that will be valid across multiple settings and countries. If these items do not work well in other settings, it may then be appropriate to consider excluding them in future validations. On the other hand, we dropped items like differential treatment, stay against will, and auditory privacy that are important to respectful maternity care and PCMC because they had low factor loadings in both samples in the initial analysis per the criteria we used. The distribution of these items likely contributed to the low loadings. Thus, even though these did not make it into the current version of the PCMC scale, they are still important to consider potentially as stand-alone questions in PCMC research as they may be more important in other settings.

We started off with several domains, which we knew to be closely interrelated, to ensure that we developed a comprehensive set of items. Thus, we expected that our items would represent a smaller number of factors than our original domains. While our factor analysis suggested a possibility of 2 to 4 sub-scales, the items did not load systematically into clean conceptual categories. This is expected given the correlation between the PCMC domains and related items. Also, the domains are overlapping rather than discrete. Thus whether “one is asked permission before procedures” is grouped under dignity and respect, communication, or autonomy is a subjective decision. This can be said of several of the items in the tool, although it is expected that there will be less disagreement as to whether these items constitute PCMC. Thus, to provide sub-scales that are practical and theory driven, we came up with the three components drawing on the experience of care domains in the WHO quality of care framework for maternal and newborn health [24]. We then examined these sub-scales in further factor analysis to ensure that only items correlated with that component were included in the sub-scale. With few exceptions, the suggested items for each sub-scale load relatively well onto the sub-scales. These sub-scales however have lower reliability (alpha between 0.6 and 0.8) than the overall 30-item scale (alpha greater than 0.8). Thus the analysis provides stronger support for a unified PCMC scale. For practical purposes, however, the sub-scales may be more useful for identifying aspects of PCMC to target for quality improvement.

We examined the PCMC scale in both a rural and urban setting to assess differences in the two contexts. It is important to note that the majority of items have strong factor loadings (>0.3) in both rural and urban settings, suggesting that this 30-item scale works relatively well in multiple contexts. Some of the items, however, work better in the rural sample, while others work better in the urban sample. One potential reason for these differences is the distribution of the variables in the different settings. For example, very few respondents in the urban reported physical abuse during their childbirth (approximately 2% said they experienced physical abuse), which could account for the poor loading with the other items. The distribution for the rural sample is slightly better, although still low; approximately 5% of respondents said they experienced physical abuse.

Another potential reason for the rural/urban difference is the differences in the sampling approaches. First, the eligibility criteria for the urban sample included only women who delivered in the preceding week, while that for rural sample included women who delivered in the preceding 9 weeks. The mean PCMC score for women who were less than 1 week postpartum is higher than that of those greater than 1 week postpartum (67 compared to 59, *p* < 0.0001). This is consistent with studies that suggest that women are less likely to report negative experiences when interviewed immediately following delivery, compared to when interviewed 5 to 10 weeks postpartum [72]. This is potentially due to social desirability bias and the joy of having just delivered a baby. Second, all interviews for the urban sample were conducted in a health facility, whereas only about 40% of the rural interviews were in a facility. The mean PCMC score for women who were interviewed in a health facility is slightly higher than that of those interviewed in the community (62 compared to 58, *p* = 0.0003). This is also unsurprising as women may not be willing to express their dissatisfaction with the care received while they are still within the health facility; they will be more comfortable talking about their experiences in their own home as opposed to a facility setting. Notwithstanding these differences in the sampling, the mean PCMC scores for the rural and urban sample was not significantly different (59 compared to 60, *p* = 0.85). This suggests that this scale may be used to measure PCMC in facility-based samples as well as community-based samples of recently delivered women up to 9 weeks postpartum, and potentially beyond.

Aside from the differences in sampling, the findings may also reflect characteristics of respondents and broader social norms in rural versus urban settings. There were small but significant differences in age, education, and marital status, with the rural women more likely to be younger, married, and with less education. These are characteristics that could affect participant responses. Moreover, expectations of care, which in turn affects satisfaction with care, [25, 28, 73] may differ between urban and rural women. In addition, the health facilities from which women were recruited in the two settings may have contributed to the findings. For example, urban facilities are typically more crowded compared to rural settings. Therefore, policies and norms around support persons in urban settings may reflect the higher volume of patients and limited space in these facilities, reflecting that a larger proportion of urban women do not want a labor and delivery support person. That the scale worked reasonably well in these different situations suggests its potential applicability in other parts of Kenya, Africa, and potentially other developing countries and regions.

Another source of heterogeneity is the different languages used in the survey. Although we took measures to ensure the meaning of the questions were similar in the different languages, nuances in language may have affected the distribution of the items. For example, the average PCMC score for Luo respondents in the rural sample is 57, compared to 62 and 63 for English and Swahili respectively. This however did not significantly affect the factor structure. Factor analysis by language of survey for the rural sample yielded 3 factors for the English and Swahili respondents and 4 factors for the Luo respondents, but in all cases, it was one dominant factor as in the main analysis.

As in any research, there are a number of limitations to this study. First, our respondents are not generalizable to all women in Kenya, and the health facilities from which women were recruited are not nationally representative. In our urban sample for example, some intervention facilities were chosen based on their willingness to participate in future quality improvement interventions. This willingness may indicate organizational readiness to change and therefore may represent higher quality facilities. Other facilities were nominated by the County, which might indicate facilities identified to have problems that could benefit from quality improvement. Additionally, all facilities in the urban area were higher-level public facilities. This was a selection criterion for the facilities to ensure delivery volumes were high enough to achieve our sample size targets. We do not know how the PCMC measures will work in smaller health centers, clinics, or private health facilities in urban Kenya. While there was no restriction for the type of delivery facility in the rural sample, interviews that occurred in health facilities were conducted in facilities selected for an intervention to improve prematurity outcomes, which included quality improvement activities. These included both hospitals and health centers, but comprised specifically of high delivery volume facilities. Thus, women who delivered in these facilities, which have been primed for quality improvement, are overrepresented in our sample. Validation of the PCMC scale in other contexts will help to assess its portability across settings.

Second, there are a number of items that have a “not applicable” response option. We included these items because they were conceptually very relevant to PCMC even if they did not apply to all respondents. Coding the not applicable category into the highest category (“all the time”) biases our results towards reporting higher levels of person-centered care. There is however no right or wrong way of addressing this challenge. If all “not applicable” responses had been coded as “missing”, approximately half of the urban sample would have been lost. Coding this category as missing on the “labor and delivery support” questions that had the largest proportion of respondents in the “not applicable” category (about 20% of combined sample), however, does not significantly change the findings. We avoided using imputations to maintain the ease of interpreting our findings, and to provide a simple roadmap for others using the scale to deal with the “not applicable” categories.

In addition, 30 items could be considered too many items in a scale. While redundancy is recommended in early stages of scale development to be inclusive, it might be possible to develop a shorter version of the scale as it is used over time across settings, and researchers are able to identify which items perform best across settings. For example, 44.7% of women in the urban setting did not want a support person during labor and 48.7% did not want a support person during delivery. While labor and delivery support is an important issue in many settings, it may not be desired by all women for a variety of reasons [69–71, 74]. Furthermore, during site visits to the facilities in urban settings, the study team became aware that labor and delivery rooms are oftentimes too crowded for women to have support persons other than facility staff in the room. We have retained delivery support, as well as crowding, in the current version of the tool, as we believe it is important for women to have a support person if so desired. With more evidence from other settings, we could shorten the scale to include only items that are relevant to the majority of women in different settings.

Moreover, while our sub-scales were both theoretically and data-driven, theory sometimes weighed more. Future validations might therefore reconsider which items fit into which sub-scale. For example, we decided to retain the health facility environment items in the SC sub-scale because the separate HFE sub-scale had low reliability, and we reasoned the HFE items are needed to provide supportive care. Most of the HFE items loaded well with other items in the SC sub-scale. There were however some items like “crowding” and “cleanliness,” which did not load well on the SC sub-scale across the settings: crowding loaded well in the urban sample, but not the rural sample, while cleanliness loaded well in the rural sample, but not urban sample. We have retained these items on the SC sub-scale as they are conceptually and empirically very relevant [75]. Future studies will help provide more empirical support for these sub-scales.

## Conclusions

This paper presents a tool for measuring PCMC in developing settings. Future studies can validate this tool to assess its appropriateness for the setting it is to be used. Where there is no capacity for validation, we believe this scale can be used to validly and reliably assess the levels of PCMC across various domains. The scale can be administered to women who have recently delivered up to 9 weeks post-partum. It can be administered through exit interviews as well as through community interviews. Ideally, people who are not considered health providers in the particular setting should conduct the interviews to reduce bias in responses. In literate populations, there is a possibility of the survey being self-administered. This scale will allow researchers to quantitatively measure women’s experiences during childbirth. In turn, this will allow comparisons across settings and time, and statistical analysis to examine the determinants and consequences of perceptions of care during childbirth. This scale can also be administered before and after interventions to improve women’s experiences during childbirth—for needs assessments as well as for monitoring and evaluation of the interventions. Facility heads and health management teams could support periodic administration of this tool to women receiving care in their facilities to assess the level of PCMC in their facilities and to identify aspects of PCMC to target for quality improvement. Developing complementary person-centered scales for other reproductive health services, such as antenatal care and family planning, will help drive the agenda to improve person-centered reproductive health care as a means of improving reproductive health outcomes.

## Additional file.

Additional file 1:
**Appendix 1.** Distribution of person-centered maternity care variables. (DOCX 126 kb)

## Abbreviations

CA: Communication and autonomy
DR: Dignity and respect
HFE: Health facility environment
KMO: Kaiser-Meyer-Olkin
PCMC: Person-centered maternity care
SC: Supportive care
WHO: World Health Organization
