# Supplementary material for: Development of a tool to measure person-centered maternity care in developing settings: validation in a rural and urban Kenyan population
Source: Reprod Health. 2017 Sep 22;14:118. doi: 10.1186/s12978-017-0381-7 (PMC5610540; doi:10.1186/s12978-017-0381-7)
Supplement: Additional file 1: — Appendix 1. Distribution of person-centered maternity care variables. (DOCX 126 kb) [file 12978_2017_381_MOESM1_ESM.docx]

| **Additional file 1: Appendix 1: Distribution of person-centered maternity care variables** | | | | | |
| --- | --- | --- | --- | --- | --- |
|  | Rural | |  | Urban | |
|  | No. | % |  | No. | % |
| How did you feel about the amount of time you waited? Would you say it was … |  |  |  |  |  |
| 0 Very short | 525 | 61.3 |  | 250 | 47.2 |
| 1 Somewhat short | 195 | 22.8 |  | 141 | 26.6 |
| 2 Somewhat long | 85 | 9.9 |  | 43 | 8.1 |
| 3 Very long | 52 | 6.1 |  | 96 | 18.1 |
|  |  |  |  |  |  |
| During your time in the health facility did the doctors, nurses, or other health care providers introduce themselves to you when they first came to see you? |  |  |  |  |  |
| 0 No, none of them | 658 | 76.8 |  | 451 | 85.1 |
| 1 Yes, a few of them | 107 | 12.5 |  | 42 | 7.9 |
| 2 Yes, most of them | 41 | 4.8 |  | 27 | 5.1 |
| 3 Yes, all of them | 51 | 6 |  | 10 | 1.9 |
|  |  |  |  |  |  |
| Did the doctors, nurses, or other health care providers call you by your name? |  |  |  |  |  |
| 0 No, never | 230 | 26.8 |  | 231 | 43.6 |
| 1 Yes, a few times | 165 | 19.3 |  | 131 | 24.7 |
| 2 Yes, most of the time | 137 | 16 |  | 94 | 17.7 |
| 3 Yes, all the time | 325 | 37.9 |  | 74 | 14 |
|  |  |  |  |  |  |
| Did the doctors, nurses, or other staff at the facility treat you with respect? |  |  |  |  |  |
| 1 Yes, a few times | 76 | 8.9 |  | 65 | 12.3 |
| 2 Yes, most of the time | 250 | 29.2 |  | 151 | 28.5 |
| 3 Yes, all the time | 514 | 60 |  | 303 | 57.2 |
|  |  |  |  |  |  |
| Did the doctors, nurses, and other staff at the facility treat you in a friendly manner? |  |  |  |  |  |
| 0 No, never | 23 | 2.7 |  | 23 | 4.3 |
| 2 Yes, most of the time | 238 | 27.8 |  | 187 | 35.3 |
| 3 Yes, all the time | 502 | 58.6 |  | 254 | 47.9 |
|  |  |  |  |  |  |
| Did the doctors, nurses, and other staff at the facility show that they cared about you? |  |  |  |  |  |
| 0 No, never | 25 | 2.9 |  | 9 | 1.7 |
| 1 Yes, a few times | 70 | 8.2 |  | 80 | 15.1 |
| 3 Yes, all the time | 507 | 59.2 |  | 236 | 44.5 |
|  |  |  |  |  |  |
| Did you feel the doctors, nurses, or other health providers shouted at you, scolded, insulted, threatened, or talked to you rudely? |  |  |  |  |  |
| 0 No, never | 763 | 89 |  | 435 | 82.1 |
| 1 Yes, once | 56 | 6.5 |  | 85 | 16 |
| 2 Yes, a few times | 22 | 2.6 |  | 10 | 1.9 |
|  |  |  |  |  |  |
| Did you feel like you were treated roughly like pushed, beaten, slapped, pinched, physically restrained, or gagged? |  |  |  |  |  |
| 0 No, never | 819 | 95.6 |  | 520 | 98.1 |
| 1 Yes, once | 24 | 2.8 |  | 10 | 1.9 |
| 2 Yes, a few times | 9 | 1.1 |  |  |  |
| 3 Yes, many times | 5 | 0.6 |  |  |  |
|  |  |  |  |  |  |
| Did you feel like you were forced to stay at the health facility against your will because you could not pay your bill? |  |  |  |  |  |
| 0 No, never | 813 | 94.9 |  | 526 | 99.2 |
| 1 Yes, for less than one day | 26 | 3 |  | 1 | 0.2 |
| 2 Yes, for one to two days | 9 | 1.1 |  | 2 | 0.4 |
| 3 Yes, for about three days or more | 9 | 1.1 |  | 1 | 0.2 |
|  |  |  |  |  |  |
| When you were speaking to the doctors, nurses or other staff at the facility, did you feel other people not involved in your care could hear what you were discussing? |  |  |  |  |  |
| 0 No, never | 547 | 63.8 |  | 414 | 78.1 |
| 1 Yes, a few times | 146 | 17 |  | 42 | 7.9 |
| 2 Yes, most of the time | 87 | 10.2 |  | 40 | 7.5 |
| 3 Yes, all the time | 77 | 9 |  | 34 | 6.4 |
|  |  |  |  |  |  |
| During examinations in the labor room, were you covered up with a cloth or blanket or screened with a curtain so that you did not feel exposed? |  |  |  |  |  |
| 0 No, never | 168 | 19.6 |  | 204 | 38.5 |
| 1 Yes, a few times | 57 | 6.7 |  | 46 | 8.7 |
| 2 Yes, most of the time | 112 | 13.1 |  | 55 | 10.4 |
| 3 Yes, all the time | 510 | 59.5 |  | 199 | 37.5 |
| 4 Not applicable | 10 | 1.2 |  | 26 | 4.9 |
|  |  |  |  |  |  |
| Do you feel like your health information was or will be kept confidential at this facility? |  |  |  |  |  |
| 0 No, never | 48 | 5.6 |  | 21 | 4 |
| 1 Yes, a few times | 105 | 12.3 |  | 58 | 10.9 |
| 2 Yes, most of the time | 263 | 30.7 |  | 132 | 24.9 |
| 3 Yes, all the time | 441 | 51.5 |  | 319 | 60.2 |
|  |  |  |  |  |  |
| Did you feel like the doctors, nurses or other staff at the facility involved you in decisions about your care? |  |  |  |  |  |
| 0 No, never | 160 | 18.7 |  | 43 | 8.1 |
| 1 Yes, a few times | 111 | 13 |  | 48 | 9.1 |
| 2 Yes, most of the time | 171 | 20 |  | 66 | 12.5 |
| 3 Yes, all the time | 344 | 40.1 |  | 236 | 44.5 |
| 4 Did not have to make any decisions | 71 | 8.3 |  | 137 | 25.8 |
|  |  |  |  |  |  |
| Did the doctors, nurses or other staff at the facility ask your permission/consent before doing procedures on you? |  |  |  |  |  |
| 0 No, never | 303 | 35.4 |  | 196 | 37 |
| 1 Yes, a few times | 118 | 13.8 |  | 80 | 15.1 |
| 2 Yes, most of the time | 195 | 22.8 |  | 130 | 24.5 |
| 3 Yes, all the time | 241 | 28.1 |  | 124 | 23.4 |
|  |  |  |  |  |  |
| During the delivery, do you feel like you were able to be in the position of your choice? |  |  |  |  |  |
| 0 No, never | 600 | 70 |  | 209 | 39.4 |
| 1 Yes, for a short time | 105 | 12.3 |  | 46 | 8.7 |
| 2 Yes, most of the time | 74 | 8.6 |  | 86 | 16.2 |
| 3 Yes, all the time | 78 | 9.1 |  | 189 | 35.7 |
|  |  |  |  |  |  |
| Did the doctors, nurses or other staff at the facility speak to you in a language you could understand? |  |  |  |  |  |
| 0 No, never | 21 | 2.5 |  | 1 | 0.2 |
| 1 Yes, a few times | 65 | 7.6 |  | 7 | 1.3 |
| 2 Yes, most of the time | 181 | 21.1 |  | 58 | 10.9 |
| 3 Yes, all the time | 590 | 68.8 |  | 464 | 87.5 |
|  |  |  |  |  |  |
| Did the doctors and nurses explain to you why they were doing examinations or procedures on you? |  |  |  |  |  |
| 0 No, never | 232 | 27.1 |  | 107 | 20.2 |
| 1 Yes, a few times | 119 | 13.9 |  | 82 | 15.5 |
| 2 Yes, most of the time | 215 | 25.1 |  | 127 | 24 |
| 3 Yes, all the time | 291 | 34 |  | 214 | 40.4 |
|  |  |  |  |  |  |
| Did the doctors and nurses explain to you why they were giving you any medicine? |  |  |  |  |  |
| 0 No, never | 141 | 16.5 |  | 94 | 17.7 |
| 1 Yes, a few times | 104 | 12.1 |  | 68 | 12.8 |
| 2 Yes, most of the time | 208 | 24.3 |  | 58 | 10.9 |
| 3 Yes, all the time | 332 | 38.7 |  | 166 | 31.3 |
| 4 Did not get any medicine | 72 | 8.4 |  | 144 | 27.2 |
|  |  |  |  |  |  |
| Did you feel you could ask the doctors, nurses or other staff at the facility any questions you had? |  |  |  |  |  |
| 0 No, never | 190 | 22.2 |  | 101 | 19.1 |
| 1 Yes, a few times | 205 | 23.9 |  | 91 | 17.2 |
| 2 Yes, most of the time | 184 | 21.5 |  | 159 | 30 |
| 3 Yes, all the time | 278 | 32.4 |  | 179 | 33.8 |
|  |  |  |  |  |  |
| Did the doctors and nurses at the facility talk to you about how you were feeling? |  |  |  |  |  |
| 0 No, never | 113 | 13.2 |  | 99 | 18.7 |
| 1 Yes, a few times | 257 | 30 |  | 110 | 20.8 |
| 2 Yes, most of the time | 220 | 25.7 |  | 193 | 36.4 |
| 3 Yes, all the time | 267 | 31.2 |  | 128 | 24.2 |
|  |  |  |  |  |  |
| Did the doctors, nurses or other staff at the facility try to understand your anxieties? |  |  |  |  |  |
| 0 No, never | 199 | 23.2 |  | 130 | 24.5 |
| 1 Yes, a few times | 199 | 23.2 |  | 59 | 11.1 |
| 2 Yes, most of the time | 146 | 17 |  | 86 | 16.2 |
| 3 Yes, all the time | 180 | 21 |  | 100 | 18.9 |
| 4 I did not have any anxieties or fears | 133 | 15.5 |  | 155 | 29.2 |
| Total | 857 | 100 |  | 530 | 100 |
|  |  |  |  |  |  |
| When you needed help, did you feel the doctors, nurses or other staff at the facility paid attention? |  |  |  |  |  |
| 0 No, never | 38 | 4.4 |  | 27 | 5.1 |
| 1 Yes, a few times | 120 | 14 |  | 99 | 18.7 |
| 2 Yes, most of the time | 324 | 37.8 |  | 246 | 46.4 |
| 3 Yes, all the time | 375 | 43.8 |  | 158 | 29.8 |
|  |  |  |  |  |  |
| Did you feel the doctors and nurses paid attention to you during your stay in the facility? |  |  |  |  |  |
| 0 No, never | 28 | 3.3 |  | 30 | 5.7 |
| 1 Yes, a few times | 129 | 15.1 |  | 100 | 18.9 |
| 2 Yes, most of the time | 336 | 39.2 |  | 232 | 43.8 |
| 3 Yes, all the time | 364 | 42.5 |  | 168 | 31.7 |
|  |  |  |  |  |  |
| Did the doctors and nurses ask how much pain you were in? |  |  |  |  |  |
| 0 No, never | 207 | 24.2 |  | 135 | 25.5 |
| 1 Yes, a few times | 213 | 24.9 |  | 117 | 22.1 |
| 2 Yes, most of the time | 204 | 23.8 |  | 162 | 30.6 |
| 3 Yes, all the time | 233 | 27.2 |  | 116 | 21.9 |
|  |  |  |  |  |  |
| Do you feel the doctors or nurses did everything they could to help control your pain? |  |  |  |  |  |
| 0 No, never | 324 | 37.8 |  | 219 | 41.3 |
| 1 Yes, a few times | 133 | 15.5 |  | 80 | 15.1 |
| 2 Yes, most of the time | 187 | 21.8 |  | 132 | 24.9 |
| 3 Yes, all the time | 213 | 24.9 |  | 99 | 18.7 |
|  |  |  |  |  |  |
| Were you allowed to eat or drink when you were hungry/thirsty? |  |  |  |  |  |
| 0 No, never | 336 | 39.2 |  | 26 | 4.9 |
| 1 Yes, a few times | 222 | 25.9 |  | 47 | 8.9 |
| 2 Yes, most of the time | 156 | 18.2 |  | 161 | 30.4 |
| 3 Yes, all the time | 143 | 16.7 |  | 287 | 54.2 |
| N/A |  |  |  | 9 | 1.7 |
|  |  |  |  |  |  |
| Were you allowed to have someone you wanted (outside of staff at the facility, such as family or friends) to stay with you during labor? |  |  |  |  |  |
| 0 No, never | 162 | 18.9 |  | 242 | 45.7 |
| 1 Yes, a few times | 103 | 12 |  | 17 | 3.2 |
| 2 Yes, most of the time | 230 | 26.8 |  | 9 | 1.7 |
| 3 Yes, all the time | 357 | 41.7 |  | 25 | 4.7 |
| 4 I did not want someone to stay with me | 5 | 0.6 |  | 237 | 44.7 |
|  |  |  |  |  |  |
| Were you allowed to have someone you wanted to stay with you during delivery? |  |  |  |  |  |
| 0 No, never | 524 | 61.1 |  | 240 | 45.3 |
| 1 Yes, a few times | 72 | 8.4 |  | 10 | 1.9 |
| 2 Yes, most of the time | 110 | 12.8 |  | 2 | 0.4 |
| 3 Yes, all the time | 138 | 16.1 |  | 20 | 3.8 |
| 4 I did not want someone to stay with me | 13 | 1.5 |  | 258 | 48.7 |
|  |  |  |  |  |  |
| Do you think there was enough health staff in the facility to care for you? |  |  |  |  |  |
| 0 No, never | 109 | 12.7 |  | 58 | 10.9 |
| 1 Yes, a few times | 119 | 13.9 |  | 77 | 14.5 |
| 2 Yes, most of the time | 250 | 29.2 |  | 198 | 37.4 |
| 3 Yes, all the time | 379 | 44.2 |  | 197 | 37.2 |
|  |  |  |  |  |  |
| Did you feel the doctors, nurses or other staff at the facility took the best care of you? |  |  |  |  |  |
| 0 No, never | 18 | 2.1 |  | 6 | 1.1 |
| 1 Yes, a few times | 77 | 9 |  | 75 | 14.2 |
| 2 Yes, most of the time | 307 | 35.8 |  | 239 | 45.1 |
| 3 Yes, all the time | 455 | 53.1 |  | 210 | 39.6 |
|  |  |  |  |  |  |
| Did you feel you could completely trust the doctors, nurses or other staff at the facility with regards to your care? |  |  |  |  |  |
| 0 No, never | 24 | 2.8 |  | 11 | 2.1 |
| 1 Yes, a few times | 84 | 9.8 |  | 60 | 11.3 |
| 2 Yes, most of the time | 293 | 34.2 |  | 171 | 32.3 |
| 3 Yes, all the time | 456 | 53.2 |  | 288 | 54.3 |
|  |  |  |  |  |  |
| During your time at the facility, did any staff at the facility ask you or your family for kitu kidogo? |  |  |  |  |  |
| 0 No, never | 791 | 92.3 |  | 529 | 99.8 |
| 1 Yes, a few times | 54 | 6.3 |  | 1 | 0.2 |
| 2 Yes, most of the time | 8 | 0.9 |  |  |  |
| 3 Yes, all the time | 4 | 0.5 |  |  |  |
|  |  |  |  |  |  |
| During your time in the health facility, would you say you were treated differently because of any personal attribute… like your age, marital status, number of children, your education, wealth, your connections with the facility, or something like that? |  |  |  |  |  |
| 0 No, never | 803 | 93.7 |  | 522 | 98.5 |
| 1 Yes, a few times | 21 | 2.5 |  | 8 | 1.5 |
| 2 Yes, most of the time | 17 | 2 |  |  |  |
| 3 Yes, all the time | 16 | 1.9 |  |  |  |
|  |  |  |  |  |  |
| Thinking about the labor and postnatal wards, did you feel the health facility was croweded? |  |  |  |  |  |
| 0 No, never | 400 | 46.7 |  | 285 | 53.8 |
| 1 Yes, a few times | 177 | 20.7 |  | 51 | 9.6 |
| 2 Yes, most of the time | 140 | 16.3 |  | 67 | 12.6 |
| 3 Yes, all the time | 140 | 16.3 |  | 127 | 24 |
|  |  |  |  |  |  |
| Thinking about the wards, washrooms and the general environment of the health facility, will you say the facility was very clean, clean, dirty, or very dirty? |  |  |  |  |  |
| 0 Very dirty | 8 | 0.9 |  | 102 | 19.2 |
| 1 Dirty | 99 | 11.6 |  | 397 | 74.9 |
| 2 Clean | 604 | 70.5 |  | 28 | 5.3 |
| 3 Very clean | 146 | 17 |  | 3 | 0.6 |
|  |  |  |  |  |  |
| Was there water in the facility? |  |  |  |  |  |
| 0 No, never | 33 | 3.9 |  | 7 | 1.3 |
| 1 Yes, a few times | 65 | 7.6 |  | 33 | 6.2 |
| 2 Yes, most of the time | 224 | 26.1 |  | 142 | 26.8 |
| 3 Yes, all the time | 535 | 62.4 |  | 348 | 65.7 |
|  |  |  |  |  |  |
| Was there electricity in the facility? |  |  |  |  |  |
| 0 No, never | 43 | 5 |  | 15 | 2.8 |
| 1 Yes, a few times | 70 | 8.2 |  | 80 | 15.1 |
| 2 Yes, most of the time | 254 | 29.6 |  | 435 | 82.1 |
| 3 Yes, all the time | 490 | 57.2 |  |  |  |
|  |  |  |  |  |  |
| In general, did you feel safe in the health facility? |  |  |  |  |  |
| 1 Yes, a few times | 57 | 6.7 |  | 34 | 6.4 |
| 2 Yes, most of the time | 198 | 23.1 |  | 91 | 17.2 |
| 3 Yes, all the time | 602 | 70.2 |  | 405 | 76.4 |
|  |  |  |  |  |  |
| Total | 857 | 100 |  | 530 | 100 |
